# Assessment of Case Fatality Rates and Overall Prevalence of Firearm Violence in California, 2005-2019

**DOI:** 10.1001/jamanetworkopen.2021.45442

**Published:** 2022-01-28

**Authors:** P. Jeffrey Brantingham, George E. Tita, Shelley Jung, Jennifer Ahern

**Affiliations:** 1 Department of Anthropology, University of California, Los Angeles; 2 Department of Criminology, Law and Society, University of California, Irvine; 3 School of Public Health, University of California, Berkeley

## Abstract

This case series uses state hospitalization and death records to examine trends in rates of gun homicides and other rates of firearm violence in California between 2005 and 2019.

## Introduction

Gun violence in the US remains a persistent public health challenge.^1^ The US gun homicide rate is nearly 25 times higher than that of any other high-income country,^2^ amounting to 13 938 deaths in 2019 alone. Although much has been made of a spike in gun violence since the onset of the global COVID-19 pandemic, it is important to note that, at least through 2019, crime and violence declined steadily for the past 3 decades. There were around 8275 fewer homicides nationally in 2019 compared with the historical peak in 1991, a 33.5% decrease.

While this decline is remarkable, it masks other concerning trends. Here we investigate the case fatality rates (CFR) of gun assaults in California, seeking to evaluate whether the total number of gun homicide incidents is higher than expected given the overall volume of gun violence.

## Methods

In this case series, we compiled data on gun violence incidents (firearm assaults and homicides) spanning 2005 to 2019 from 2 sources: emergency department and inpatient hospitalization discharge records from the California Department of Health Care Access and Information, and death records from the California Department of Public Health Vital Records. Records included all hospital visits and deaths in California, with the exception of active-duty military hospitals, and captured the external cause of death or injury. People who went to the emergency department before being admitted to a hospital only had a hospitalization record. People who died without seeking care or after being released from care at an emergency department or hospital were only included from the death records. Thus, there were no duplicates of incidents incorporated in the measures.

We identified gun assaults from the emergency department and inpatient hospitalization data using *International Classification of Diseases, Ninth Revision, Clinical Modification* (*ICD-9-CM*) codes.^3^ We identified gun homicides from the death records using *International Statistical Classification of Diseases and Related Health Problems, Tenth Revision *(*ICD-10*) codes.^4^ We compiled annual counts of gun assaults and homicides and then computed the rate of firearm injury per 100 000 population per year and the CFR per year based on the proportion of the total shootings that resulted in fatalities. We examined statewide trends using linear regressions implemented in Stata version 17.0 (StataCorp). Our results follow the Strengthening the Reporting of Observational Studies in Epidemiology (STROBE) reporting guideline. This study was reviewed and approved by the California Health and Human Services Agency Committee for the Protection of Human Subjects and the University of California, Berkeley institutional review board, and was exempted from informed consent requirements because deidentified data were used.

## Results

The number of nonfatal gun assaults (6787 in 2005 vs 3803 in 2019), number of gun homicides (1932 in 2005 vs 1282 in 2019), and gun injury rate per 100 000 population (24.3 deaths in 2005 vs 12.9 deaths in 2019) all declined significantly between 2005 and 2019 (Figure; Table). The CFR increased significantly during the study period, from 22.2% in 2005 (1932 homicides out of 8719 gun crimes) to 25.2% in 2019 (1282 homicides of 5805 gun crimes) (Table). Holding the CFR steady at the 2005 rate estimated from the linear model (20.8%) and assuming that total volume of shootings remained as observed, we calculated an excess mortality of 1919 gun homicides in California over the subsequent 14 years (ie, 2006-2019) associated with the increase in CFR alone.

**Figure.**
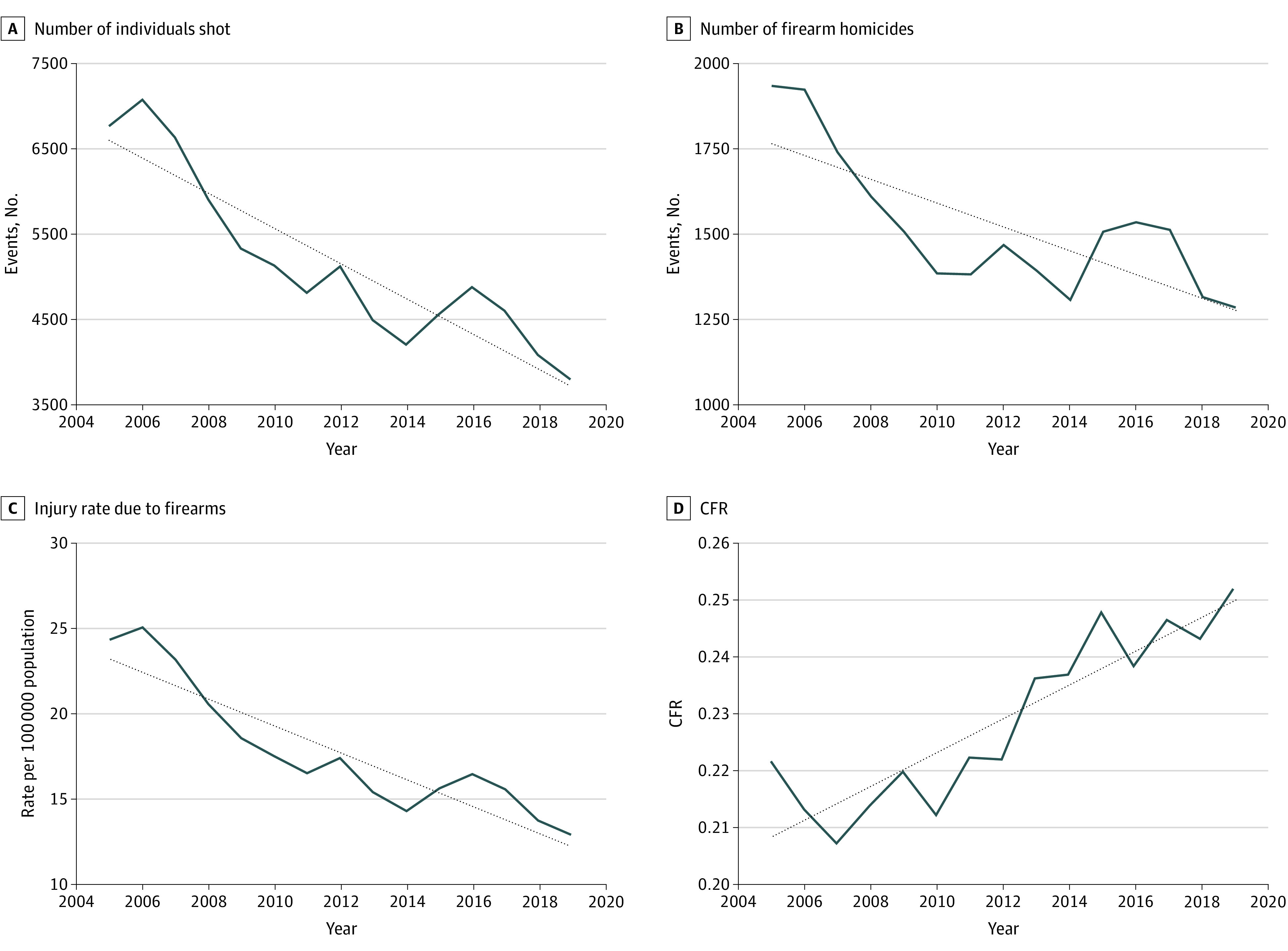
Rates of Injury and Homicide Associated with Firearms CFR indicates case fatality rate.

**Table.** Ordinary Least Squares Regression Diagnostics for Nonfatal Gun Assaults, Gun Homicides, Gun Violence Rates, and Case Fatality Rate, 2005-2019

Slope and intercept	Coefficient (95% CI)^a^	SE	*t*	*P* value
Nonfatal gun assault				
β1	–206.3 (–258.5 to –154.1)	24.1	–8.5	<.001
β0	420 225.2 (315 224.3 to 525 226.0)	48 603.2	8.7	<.001
Gun homicide				
β1	–35.0 (–52.4 to –17.6)	8.1	–4.3	.001
β0	71 866.7 (36 847.7 to 106 885.6)	16 209.7	4.4	.001
Gun violence				
β1	–0.8 (–1.0 to –0.6)	0.1	–8.6	<.001
β0	1590.5 (1196.6 to 1984.3)	182.3	8.7	<.001
Case fatality rate				
β1	0.003 (0.002 to 0.004)	0.0004	6.8	<.001
β0	–5.8 (–7.7 to −3.9)	0.9	–6.6	<.001

^a^ Computed using Stata version 17.0 regress command with robust standard errors.

## Discussion

In this study, the number of nonfatal gun assaults in California decreased more quickly than the number of gun homicides. The trend is consistent with patterns observed in trauma center data.^5^ The ultimate cause of the trend requires further investigation, but it may be related to gradual changes in the caliber of guns in circulation.^6^

Our study is limited by a lack of situational details about individual crimes that would be necessary to evaluate this hypothesis. Nevertheless, the shift in CFR is practically significant.
